# Measures for Controlling Gaseous Emissions during Composting: A Review

**DOI:** 10.3390/ijerph20043587

**Published:** 2023-02-17

**Authors:** Minghan Li, Shuyan Li, Shigeng Chen, Qingyu Meng, Yu Wang, Wujie Yang, Lianhui Shi, Fangjun Ding, Jun Zhu, Ronghui Ma, Xinsong Guo

**Affiliations:** 1 College of Resource and Environment, National Engineering Laboratory for Efficient Utilization of Soil and Fertilizer Resources, Shandong Agricultural University, Tai’an 271018, China; 2 SDAU Fertilizer Science & Technology Co., Ltd., Tai’an 271608, China; 3 Shandong Agricultural Technology Extension Center, Jinan 250014, China

**Keywords:** composting, greenhouse gaseous, odors, additives

## Abstract

Composting is a promising technology for treating organic solid waste. However, greenhouse gases (methane and nitrous oxide) and odor emissions (ammonia, hydrogen sulfide, etc.) during composting are practically unavoidable, leading to severe environmental problems and poor final compost products. The optimization of composting conditions and the application of additives have been considered to mitigate these problems, but a comprehensive analysis of the influence of these methods on gaseous emissions during composting is lacking. Thus, this review summarizes the influence of composting conditions and different additives on gaseous emissions, and the cost of each measure is approximately evaluated. Aerobic conditions can be achieved by appropriate process conditions, so the contents of CH_4_ and N_2_O can subsequently be effectively reduced. Physical additives are effective regulators to control anaerobic gaseous emissions, having a large specific surface area and great adsorption performance. Chemical additives significantly reduce gaseous emissions, but their side effects on compost application must be eliminated. The auxiliary effect of microbial agents is not absolute, but is closely related to the dosage and environmental conditions of compost. Compound additives can reduce gaseous emissions more efficiently than single additives. However, further study is required to assess the economic viability of additives to promote their large-scale utilization during composting.

## 1. Introduction

Urbanization, driven by global scientific and technological progress and economic development, has led to increased utilization of produced solid waste [1,2]. Classified according to its source, solid waste includes household waste produced by human activities, poultry manure produced by livestock breeding, dewatered sludge produced by sewage treatment, and other types of waste produced by agriculture, industry, and garden forestry. Among these, the daily disposal volume of municipal solid waste (MSW) is huge and constantly growing. According to the National Bureau of Statistics of the People’s Republic of China [3], the annual volume of MSW in 2020 sharply increased to 235.12 million tons, which is an increase of 1.03% compared with the same period in 2019, and the harmless disposal rate reached 99% [3]. There is also annual production of 3.8 billion tons of poultry manure, which introduces a big problem in the harmless disposal of solid waste [4]. For clean production and sustainable development, the composting method has been extensively studied to improve safety and waste utilization efficiency. Composting studies indicate incomparable advantages over traditional solid waste disposal, both in laboratory experiments and real production, and it is especially suitable for the utilization of the perishable components of livestock, sewage sludge, and household waste [5,6,7]. However, although composting fulfills the fertilizer requirements for soil and crops, it also leads to many problems, especially regarding gaseous emissions. Untreated solid waste produces a large amount of GHGs, but even with composting treatment, a small amount of GHGs are emitted during composting or are released after application, posing a potential threat to the climate and atmosphere [8,9]. As an organic fertilizer, compost needs to provide sufficient nutrients to crops. However, gaseous emissions during composting cause the loss of carbon, nitrogen, and sulfur nutrients [10]. According to previous studies, ammonia (NH_3_) and nitrous oxide (N_2_O) contribute 79–94% and 9.2–9.9% to total nitrogen loss, respectively [11]. Meanwhile, methane (CH_4_) emissions during composting lead to approximately 1.85% total carbon loss [12]. The major contributor to global warming potential (GWP) is N_2_O emissions (81.44–95.02%), followed by CH_4_ (0.82–7.14%) and NH_3_ (3.80–11.42%) emissions, indicating that N_2_O emissions are several times more critical for global warming than those of other gases [13]. Nowadays, GHG emissions have led to severe global warming, abnormal crustal movement, and impacts on biological activities, which are not neglectable [14,15]. To further improve the feasibility of safe composting and expand the production scale, it is necessary to take corresponding measures to mitigate GHG emissions. Additionally, the process of composting is accompanied by an unpleasant odor, which mainly originates from the volatile compounds containing sulfur and nitrogen [16,17]. Therefore, the construction of composting equipment and facilities should be improved to regulate the compost production conditions more strictly.

Previous studies confirmed that an improved aerobic fermentation process and composting additives can significantly reduce GHGs and odorous gaseous emissions. The inadequate physical properties of compost are among the primary factors leading to gaseous emissions. Adding organic and mineral materials can significantly enhance the pore structure of compost by increasing its specific surface area [18]. With a reduction in the anaerobic area, the loss of nutrients caused by the evaporation of CH_4_ and other gaseous products is significantly lower [19,20]. Li et al. [21] used biochar and electric field-assisted composting to reduce GHGs by 31.6%, implying that combining measures and additives is very attractive for controlling gaseous emissions from composting. Adjusting the chemical environment and inoculating microbial agents can also promote compost maturation and reduce the emissions of related gases [22,23]. Yang et al. [24] demonstrated that the combination of dicyandiamide and phosphogypsum significantly reduced GHGs by 37.46%, demonstrating the great potential of using combined treatments in reducing gaseous emissions from composting. Furthermore, research on the influence of other novel, low-cost, and highly efficient additives and compound additives has certainly become one of the important future research directions. Therefore, using additives or other measures can enable the effective control of gaseous emissions during composting, representing a research hotspot toward improved environmental benefits from composting. Meanwhile, the development of composting must conform to stricter legal regulations, which requires gaseous emissions from composting to strictly meet the requirements of cleaner production to minimize the impact on global warming. Under this situation, it is urgent to review the efficiency of measures implemented to control gaseous emissions from composting as a scientific topic.

This review article focuses on the control strategies of GHGs and odorous gaseous emissions in the recent research literature. Based on the treatment process characteristics and the principle of minimizing gaseous emissions, the additives in this paper are divided according to the process conditions into physical, chemical, microbial, and compound additives. The advantages and disadvantages of additives are also reviewed. This review aims to provide a comprehensive analysis of gaseous emission control strategies during composting, summarize the current research results, and propose future research directions.

## 2. The Theory of Gaseous Emission in Composting

In the composting process, organic matter in solid waste is mineralized by microorganisms, and organic nitrogen is transformed into ammonium nitrogen, which mainly escapes in the form of NH_3_. Organic carbon is decomposed to provide energy for microbial activities and mainly escapes in the form of carbon dioxide (CO_2_). Sulfur-containing organics decompose and disperse in the form of dimethyl sulfide (Me_2_S), dimethyl disulfide (Me_2_SS), etc. [25]. In addition to the above gaseous emissions, there are small amounts of N_2_O and CH_4_ emitted. Excessive gaseous emissions during composting may affect the efficiency of the compost as a fertilizer. For the atmospheric environment, the aerobic composting process is dominated by vigorous microbial activities and continuous GHG output, causing severe air pollution.

**Figure 1:**
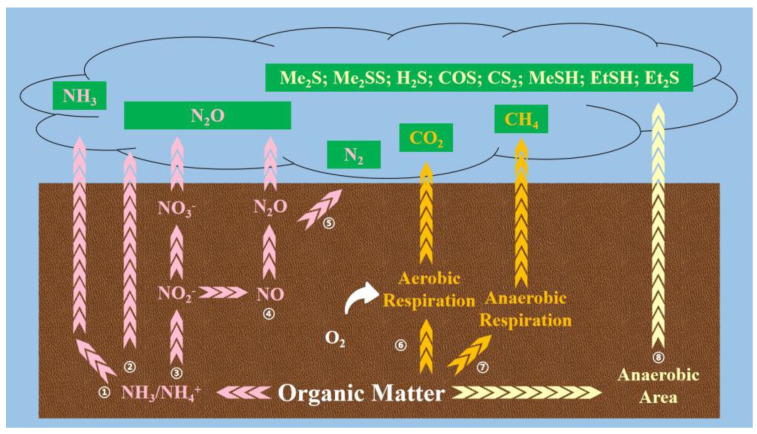
Conversion process of gaseous emissions during composting.

In this review, the gaseous emissions of composting are classified into three types: nitrogenous, carbonaceous, and sulfurous gaseous emissions. The theory of each element is discussed as follows:

1. Nitrogen transformation and gaseous emissions
  (1) The metabolic pathway of NH_3_: First, nitrogenous organic compounds from solid waste are mineralized into NH_4_^+^_,_ and a small amount of NH_3_ is directly produced by microorganisms [26]. Caused by the rising temperature of the composting pile, highly unstable NH_4_^+^ continues to transform into NH_3_ (pathway ①) [27];
  (2) The metabolic pathway of N_2_O: The generation of N_2_O occurs via three pathways, as shown in Figure 1. First, under ammonification driven by ammonia-oxidizing bacteria, hydroxylamine is generated from NH_4_^+^ by ammonia monooxygenase as an intermediate product. After that, hydroxylamine is transformed into NO_2_^−^ by hydroxylamine oxidoreductase [28,29]. The remaining NH_4_^+^ directly generates N_2_O by incomplete nitrification (pathway ②) [30]. Second, with NO_2_^−^ oxidized to NO_3_^−^ by nitrite oxidoreductase, incomplete denitrification transforms a part of the NO_3_^−^ into N_2_O. A small amount of NO_3_^−^ is reduced to NO_2_^−^ by the nitrate reductase (pathway ③) [31]. Third, denitrifying bacteria transform the rest of the NO_2_^−^ into NO by nitrite reductase and further convert it to N_2_O by nitric oxide reductase (pathway ④) [32];
  (3) With the above N_2_O emitted into the air, the rest is completely denitrified to N_2_ by nitrous oxide reductase, so the nitrogen metabolic pathway during composting is over (pathway ⑤) [33].

2. Carbon transformation and gaseous emissions
  The major carbon loss in compost originates from CO_2_ produced by aerobic decomposition and respiration by microbes, but this carbon loss is necessary for microbial activity (pathway ⑥) [34]. Compared with CO_2_, CH_4_ represents a more severe, but controllable, threat to global warming [8,35]. As mineralization proceeds, the composting pile continuously shrinks and compacts, creating more anaerobic areas [36]. In this situation, the activity and propagation of methanogens are improved, so more CH_4_ is produced from the composting pile (pathway ⑦) [37].

3. Sulfur transformation and gaseous emissions
  Volatile sulfide compounds (VSCs) also result from the formation of anaerobic areas in compost. Therefore, odor generation can be used as qualitative proof of poor physical properties [38]. The degradation of sulfur-containing amino acids under anaerobic conditions and the methylation of hydrogen sulfide (H_2_S) or methyl mercaptan (MeSH) lead to the emission of odorous gases (pathway ⑧), severely affecting human health and the compost’s fertility [39]. VSCs include Me_2_S, Me_2_SS, H_2_S, MeSH, ethyl mercaptan (EtSH), diethyl sulfide (Et_2_S), carbonyl sulfide (COS), carbon disulfide (CS_2_), etc. [40], and the emissions of Me_2_S and Me_2_SS may especially lead to strong sulfur loss with an unpleasant smell [41,42].

## 3. Control Strategy for Gaseous Emissions

### 3.1. Composting Process Conditions

#### 3.1.1. Efficiency Analysis

As shown in Table 1, improvements in the process conditions were made to provide accurate and effective composting control; GHGs and odorous gaseous emissions can also be reduced.

The aeration mode has a decisive influence on the composting process. As a crucial process parameter, a higher aeration rate can greatly reduce the anaerobic area, but it faces stronger NH_3_ emissions and temperature loss [43]. Conversely, lower aeration rates can cause anaerobic, incomplete nitrification, and incomplete denitrification reactions, leading to the production of GHGs and odors [44]. Therefore, as one of the most important composting parameters, it is necessary to have an appropriate aeration rate and method. An intermittent aeration rate of 0.3–0.5 L/min/kg DM has been reported to be a suitable aeration method [45]. Xu et al. [46] adopted an aeration rate of 0.48 L/min/kg DM for kitchen and garden waste co-composting. Compared with a treatment at a lower aeration rate, the experimental results showed that the aeration rate of 0.48 L/min/kg DM significantly reduced the emissions of CH_4_, N_2_O, and H_2_S. It was demonstrated that a higher aeration rate inhibited the expression of functional genes related to GHGs and sulfurous odors emission. Thus, excessive GHGs and sulfurous odor emissions can be reduced [47]. Negative pressure aeration is a novel technology based on traditional passive aeration, where the oxygen supply depends on the temperature gap [48]. Wang et al. [49] observed that a negative pressure aeration rate of 0.75 L/min/kg DM reduced NH_3_ volatilization by 55%, accompanied by small increases in CH_4_ and N_2_O emissions. Compared with passive aeration, negative pressure changed the airflow direction and captured more ammonium nitrogen in the composting pile [50]. Although CH_4_ and N_2_O emissions were slightly increased, negative pressure was more beneficial to reduce the total GHG emissions at the same aeration rate [49]. Compared with continuous aeration, intermittent aeration is more helpful for maintaining a constant temperature and reducing gaseous emissions [51]. According to the research by Ma et al. [52], an aeration interval of 30 min on–30 min off decreased CH_4_ and N_2_O emissions by 9.68% and 47.10%, respectively. Compared with treatments with an interval time of less than 30 min/h, 30–30 intermittent aeration was more detrimental to pore retention. Under such airflow conditions, the anaerobic area was greatly reduced, and CH_4_ and N_2_O emissions were also effectively inhibited [53]. In conclusion, the formulation of a specific experimental aeration method still needs to be adjusted according to the material and pre-experimental results, based on the existing research.

Membrane composting is a relatively mature technology that can effectively reduce air pollution caused by composting [54]. Xiong et al. [55] observed that applying the functional membrane-covering technique (FMCT) can reduce N_2_O emission by 16.44–41.15% because the FMCT fixes the inner pressure to the micro-positive pressure, maintaining an appropriate temperature while ensuring aerobic conditions and oxygen utilization efficiency. Nitrifying bacteria are extremely sensitive to high temperatures, so denitrification and N_2_O production may be simultaneously significantly inhibited [56]. Even so, the FCMT increased NH_3_ emissions by 13.78–73.37%. During the thermophilic period, the FMCT treatment exhibited a more intense degree of mineralization, leading to the accumulation of NH_4_^+^/NH_3_. The experimental data showed that the temperature and pH of the FMCT treatment were higher, causing stronger evaporation and NH_3_ emissions [57]. Sun et al. [58] and Fang et al. [59] performed similar research on semi-permeable membrane-covered hyperthermophilic composting (smHTC). The results showed that smHTC significantly reduced the CH_4_ and N_2_O emissions, especially in the thermophilic phase. Compared with the common method, smHTC suppressed the expression of *mcrA* by 1.6 times, which is the key functional gene related to CH_4_ emissions and oxidation [58]. In another dairy manure-composting experiment, smHTC reduced the CH_4_ and N_2_O emissions by 99.89% and 60.48% during the aeration interval, respectively [59]. The positive micro-pressure and aerobic environment facilitated oxygen permeation and utilization by microorganisms, which was created by smHTC. The high-temperature environment in the membrane intensified water volatilization, condensing a water layer close to the membrane, which quickly blocked part of the gaseous volatilization. However, with a decrease in humidity, the interception effect of the water layer decreased. The dissolved NH_4_^+^ was converted into NH_3_ and re-released, which explained the sudden increase in the NH_3_ emissions later. Compared with the inside gaseous emissions, the outside emissions were easier to control. This indicates that the correction of NH_3_ emissions was limited and needed to be combined with other additives.

In addition, electric field-assisted composting is a recent research hotspot. By applying a 2 V direct-current electric field to the composting pile, the reproduction of electroactive bacteria was promoted, and oxygen utilization was improved [60]. The driving effect of the electric field on the ions accelerated the compost’s maturation, promoted microbial activity, and produced more heat. An environment dominated by an electric field and high temperature can inhibit the activity of denitrifying bacteria, promote oxygen uptake, and reduce the production of N_2_O and CH_4_ [21,60]. Combining electric field composting technology with additives and further exploring the influence of the direct-current electric field on nitrogen fixation, ammonification, nitrification, and denitrification are future research directions.

**Table 1:** The effects of process conditions on GHGs and odors during composting.

Feedstock	Measure	Impact on Gaseous Emissions (Relative to Control)				Note	Reference
		CH_4_	N_2_O	NH_3_	VSCs		
Cow manure, wheat straw	Functional membrane-covered composting		−16%	+14%		Lasted for 36 days; promoted temperature rise	[55]
Chicken manure, mushroom residue, crop stalk, bran	Semi-permeable membrane-covered composting	−79%	−45%			Lasted for 24 days; reduced emissions based on thermophilic phase; promoted temperature rise; initial C/N: 24	[58]
Cow manure	Semi-permeable membrane-covered composting	−100%	−61%			Lasted for 30 days; promoted temperature rise; initial C/N: 34	[59]
Kitchen waste, garden waste	Aeration intensity (0.48 L/kg/min) ^2^	↓	↓	↑	↓	Lasted for 35 days; suppressed temperature rise	[46]
Cow manure, corn stalk	Negative pressure aeration (0.75 L/min/kg) ^2^	↑	↑	↓		Lasted for 35 days; decreased electrical conductivity	[49]
Chicken manure, mushroom residue	Intermittent aeration (10 min on–30 min off)	↑	↑			Lasted for 36 days; suppressed temperature rise; reduced emissions based on outside of membrane; initial C/N: 34	[52]
Chicken manure, rice husk	Electric field-assisted composting (2 V DC)	↓	↓			Lasted for 30 days; promoted temperature rise; increased electrical conductivity	[21]
Chicken manure, mature compost, rice husk, dewatered sewage sludge	Electric field-assisted composting (2 V DC)	↓	−73%			Lasted for 30 days; promoted temperature rise	[60]

Note: ^2^ dry weight basis; ↑: increase (no detailed data); ↓: decrease (no detailed data); VSCs: volatile sulfide compounds.

#### 3.1.2. Cost Assessment and Economic Benefits

Although advanced process conditions can effectively alleviate gaseous emissions during composting, the related high costs cannot be ignored. Therefore, the above process conditions are more presented to provide new ideas for the construction of basic composting facilities. After stable and efficient process conditions are determined, it is necessary to continue to reduce the equipment cost in large-scale production and evaluate its economic benefits.

### 3.2. Physical Additives

According to previous research studies, physical additives are mainly used for preserving the pore structure of a composting pile and can be subdivided into organic physical additives and mineral physical additives [61].

#### 3.2.1. Organic Physical Additives

Due to its large specific surface area and low cost, biochar has been widely studied as a physical additive in composting [29,62]. As shown in Table 2, biochar can be produced from bamboo, corn stalk, wheat straw, willow chips, and even poultry manure [19,20,63]. As reported by Zhang et al. [64], bamboo biochar exerted a considerable effect on the reduction in gaseous emissions during composting, decreasing the emissions of GHGs and NH_3_ by 93.61% and 51.42%, respectively. Adding biochar can greatly inhibit the formation of an anaerobic area in a composting pile. As the efficiency of oxygen utilization improves, methanogenesis and incomplete denitrification are inhibited, thus reducing methane and nitrous oxide emissions [65]. Biochar can also regulate the activities of urease, catalase, and other key enzymes in compost, accelerate decomposition, and better fix the volatile ammonium nitrogen in the pores [66]. Additionally, biochar has the same correction effect for sulfur-containing odor emissions. Liu et al. [67] proved that biochar significantly mitigated H_2_S, Me_2_S, and Me_2_SS emissions. By adding biochar to avoid the formation of an anaerobic area in a composting pile to the largest possible extent, odors produced by sulfur-containing amino acids are greatly reduced [68]. The use of apple pomace, wood vinegar, and other organic additives discarded as waste has also been reported [22,67,69,70]. In particular, additives such as apple pomace and furfural residue can neutralize an alkaline environment, inhibiting the shift in the chemical equilibrium toward the formation of volatile NH_3_ [71]. These additives have multiple advantages because their use enables, at the same time, a feasible way to recycle waste and a great gaseous emission reduction effect.

#### 3.2.2. Mineral Physical Additives

As shown in Table 2, mineral additives, such as clay, medicinal stone, zeolite, diatomite, and bentonite, have mainly been used in previous studies [18,69]. Like organic additives, mineral additives provide a sufficient specific surface area and a suitable micropore structure, which are beneficial for oxygen diffusion and microbial activity. In addition, functional groups in minerals can reduce the volatilization of related gases through their complexation and ion exchange [72,73]. By employing diatomite, Ren et al. [74] reduced NH_3_, N_2_O, and CH_4_ emissions by 23.70%, 84.16%, and 30.41%, respectively. The absorption capacity of microporous crystals could restrain the formation of an anaerobic area, and ions, such as NH_4_^+^, could be adsorbed simultaneously [75]. In particular, spectral data indicated that mineral additives can accelerate the humification of compost [76]. The formation of aromatic functional groups can reduce nutrient loss through a complexation reaction, thus controlling GHG emissions. Wang et al. [18] reported that adding zeolite, medical stone, and Ca-bentonite increased NH_3_ emissions while reducing N_2_O and CH_4_ emissions. Furthermore, although the oxygen supply efficiency was improved, the airflow carried more NH_3_ into the air [77]. Hence, the additive amount needs to be moderate to minimize GHG emissions.

**Table 2:** The effects of physical additives on GHGs and odors during composting.

Feedstock	Additive	Impact on Gaseous Emissions (Relative to Control)				Note	Reference
		CH_4_	N_2_O	NH_3_	VSCs		
Pig manure, wheat straw	10% bamboo biochar ^1^		↓	↓		Lasted for 27 days; suppressed temperature rise	[29]
Sewage sludge, straw	5% bamboo biochar ^1^	−16%	−5%			Lasted for 29 days; promoted temperature rise	[19]
Chicken manure, tobacco waste	10% bamboo biochar ^2^	↓	−94%	−51%		Lasted for 35 days; promoted temperature rise; decreased electrical conductivity	[64]
Pig manure, corn stalk	10% spent mushroom substrate ^2^		−37%	−7%	↓	Lasted for 49 days; promoted temperature rise; H_2_S: –13%, –33%; Me_2_S and Me_2_SS: both more than –50%	[67]
	10% straw biochar ^2^		↑	−24%	↓		
Pig manure, sawdust	10% clay ^2^	−46%	−87%			Lasted for 42 days; promoted temperature rise; initial C/N: 35	[76]
Chicken manure, caraganna microphylla straw	12.5% gasification filter cake ^2^			↓		Lasted for 50 days; promoted temperature rise; initial C/N: 43	[78]
Pig manure, wheat straw	10% fine coal gasification slag ^2^	−72%	−77%	−28%		Lasted for 42 days; promoted temperature rise; initial C/N: 29	[70]
Pig manure, sawdust	5% medical stone ^2^	↓	↓	↓		Lasted for 36 days; promoted temperature rise	[69]
	5% zeolite ^2^	↓	↓				
	2% wood vinegar ^2^	↓	↓	↓			
Pig manure, sawdust	10% diatomite ^2^	−30%	−84%	−24%		Lasted for 42 days; promoted temperature rise	[74]
Dewatered sewage sludge, wheat straw	5% apple pomace ^2^	−22%	−33%	↓		Lasted for 45 days; similar temperature variation	[22]
Poultry manure, sugar cane straw, mature compost	10% green waste biochar ^2^	↓	↓			Lasted for 60 days; similar temperature variation; both reduced CH_4_ and N_2_O significantly	[63]
	10% poultry litter biochar ^2^	↓	↓				
Sewage sludge, wheat straw	10% zeolite ^2^	−88%	−84%	↑		Lasted for 56 days; promoted temperature rise; Ca-bentonite led to higher NH_3_ emissions	[18]
	10% Ca-bentonite ^2^	−86%	−81%	↑			
	10% medical stone ^2^	−87%	−80%	↑			
Poultry manure, wheat straw	10% willow woodchips biochar ^1^			↓		Lasted for 42 days; promoted temperature rise; NH_3_ emissions reduced by over 50%	[62]
Cattle manure, rice straw	3% straw biochar (*w/v*)		−54%			Lasted for 65 days; promoted temperature rise	[79]

Note: ^1^ wet weight basis; ^2^ dry weight basis; ↑: increase (no detailed data); ↓: decrease (no detailed data); VSCs: volatile sulfide compounds.

#### 3.2.3. Cost Assessment and Economic Benefits

Most of the physical additives reviewed above are recycled waste, highlighting their favorable, low-cost advantages. However, plausible future large-scale production needs a continuous and stable supply source. These problems are major challenges for physical additives and cost factors that must be pre-evaluated for large-scale operations.

### 3.3. Chemical Additives

As shown in Table 3, chemical additives can effectively reduce NH_3_ emissions during composting. On one hand, chemical additives, such as acids, mitigate NH_3_ emissions by adjusting the pH, which can neutralize the alkaline environment and inhibit the transformation of NH_4_+ to NH_3_ [80]. On the other hand, crystallization and precipitation methods can strengthen nitrogen retention to reduce NH_3_ emissions [81]. By adding chemical additives, extra nutrients are provided, and the pore structure inside precipitates is conducive to material exchange and microbial attachment [82,83]. The statistics in Table 3 show that other GHGs and the odor reduction performance of chemical additives are not as significant as those for NH_3_. Therefore, the action mechanism for other GHGs and odors should be analyzed under specific conditions.

#### 3.3.1. pH Adjustment

As the main source of nitrogen loss in composting, NH_3_ emissions are concentrated in the thermophilic period [76,84]. With the violent mineralization of organic matter, the content of ammonium nitrogen increases and is transformed into ammonia (as shown in Figure 1). Alkaline environments, high temperatures, and low moisture contents are unfavorable for the fixation of NH_3_, while adjusting the pH via acidic substances is an effective strategy [20,85]. Nie et al. [2] reported that adding 1% lactic acid (on a dry weight basis) reduced nitrogen loss from NH_3_ emissions by 14.65% and increased the relative abundance of lactic acid bacteria. Cao et al. [71] used sulfuric acid to adjust the compost’s pH to 6. After acidification, the conversion of ammonium to NH_3_ was inhibited, and NH_3_ volatilization from the compost was significantly mitigated. Referring to the results of Pan et al. (2018), adding 1% citric acid or 3% phosphoric acid (on a dry weight basis for both) to compost could also alleviate the emissions of NH_3_. Salts, such as MgCl_2_ and FeSO_4_, were found to reduce NH_3_ emissions by 58.3% and 82.9%, respectively [85]. Therefore, it has been experimentally confirmed that organic/mineral acids and acidic salts can effectively reduce NH_3_ emissions.

However, the influence of acid additives on GHGs is variable. As a strong mineral acid, the addition of sulfuric acid reduces the pH and inoculates the composting pile with SO_4_^2−^, alleviating CH_4_ emissions [71]. Like the addition of sulfur powder, the sulfide concentration in the compost remains high after participating in microbial metabolism. Pan et al. (2018) reported that 70.57% of CH_4_ emissions were mitigated after sulfur addition. Thus, it can be confirmed that a high sulfate concentration can reduce CH_4_ emissions by changing the redox potential and inhibiting the growth of methanogens [86,87,88]. A high concentration of ammonium nitrogen also has an inhibitory effect on methanogens, so more ammonia can be fixed under the action of acidic additives, and CH_4_ production can be further reduced [89]. Although low pH values may stimulate N_2_O emissions, Pan et al. [22] found that citric and phosphoric acids differently affected N_2_O emissions. The related statistical data showed that citric acid reduced N_2_O emissions by 51.26%, but phosphoric acid increased N_2_O emissions by 31.89%. The emissions of CH_4_ and N_2_O are mainly generated by an anaerobic area and incomplete nitrification/denitrification [90]. Ren et al. [13] reported that a high dose of sodium selenite reduced N_2_O emissions by 30.45–69.54%. In contrast, Wang et al. [91] observed that adding sodium selenite increased N_2_O emissions by 29.7%, while sodium selenate reduced them by 69.3%. Adding sodium selenite and sodium selenate increased CH_4_ emissions by 18.4% and 3.5%, respectively. According to the study by Li et al. [85], the addition of salts also had no significant correction effect on GHGs. Due to the differences in raw materials and composting conditions between various studies, different microbial communities exhibited different tolerance levels to acid addition and the formation mechanism of anaerobic areas [92]. Thus, it can be concluded that organic/mineral acids and salts have no significant correction effect on N_2_O and CH_4_ emissions.

The addition of salts can also reduce gaseous emissions through redox reactions. Previous studies proved that strong oxidants, such as hypochlorite and permanganate, can reduce NH_3_ emissions by advancing nitrification [27,85]. Under the action of a strong oxidant, nitrogen is fixed as nitrate nitrogen, which is more stable [93]. Moreover, some studies also indicated that Fe_2_O_3_ has satisfactory performance in reducing the volatilization of sulfur-containing odors [17]. With Fe_2_O_3_ correction, the emissions of volatile sulfur compounds, such as COS, CS_2_, MeSH, and Me_2_SS, were reduced by 46.7–80.8%, but this still needs to be verified in further research.

#### 3.3.2. Struvite Crystallization

Struvite crystallization has become a novel method to increase nitrogen stabilization, and it is used in agriculture as a slow-release fertilizer [40]. The main component of struvite crystallization is magnesium ammonium phosphate hexahydrate, which is mainly produced by the reaction of NH_4_^+^, Mg^2+^, and PO_4_^3−^ in compost [94,95]. HPO_4_^2−^ and H_2_PO_4_^−^ species derived from the hydrolysis of PO_4_^3-^ continue to react with the above substrates to generate H^+^, the environment of a composting pile is buffered, and the ammonium nitrogen can be fixed [73]. As shown in Table 3, Jiang et al. [81] used several different “magnesium salt + phosphate additive” combinations and verified that struvite crystallization effectively improved nitrogen fixation and decreased related gaseous emissions. Furthermore, Zhang et al. [83] found that adding calcium superphosphate reduced NH_3_, CH_4_, and H_2_S by 37.9%, 35.5%, and 65.5%, respectively; moreover, the emissions of N_2_O were also stronger, demonstrating the same result as Pan et al. [22]. However, Yuan et al. [82] and Zhang et al. [64] showed that N_2_O emissions were reduced. Like the addition of magnesium salt and phosphate additives, calcium superphosphate participates in struvite crystallization via microbial metabolism. As the struvite crystallization process proceeds, free NH_4_^+^ in a composting pile exists more in a stable crystalline form and the precursor of NH_3_ generation is controlled [96]. Previous studies confirmed that a high concentration of NH_4_^+^ can inhibit the reproduction of methanogens and methane metabolism [89]. Additionally, crystallization is helpful to maintain oxygen supply pores. According to the same mechanism, phosphogypsum can effectively reduce NH_3_ and CH_4_ emissions during composting, and the SO_4_^2−^ component of phosphogypsum provides an additional effect for inhibiting the activity of methanogens [71,82,97]. The modification of the anaerobic area by struvite crystallization is also beneficial for controlling the emissions of sulfur odors [83]. Therefore, struvite crystallization can significantly mitigate NH_3_ and CH_4_ emissions and enhance nitrogen fixation and oxygen supply efficiency, while the effect of struvite crystallization on N_2_O emission is not significant [64,81,83].

#### 3.3.3. Dicyandiamide

As a nitrification inhibitor, it was confirmed that dicyandiamide (DCD) could inhibit the metabolism of ammonia-oxidizing bacteria and control the emissions of N_2_O [98,99]. The addition of DCD could reduce the emissions of NH_3_, N_2_O, and CH_4_ by 9.37%, 31.79%, and 9.6%, respectively [24]. DCD was found to prevent the conversion of ammonium nitrogen to nitrite nitrogen while maintaining a high concentration of ammonium nitrogen in the system, so the emissions of NH_3_ and CH_4_ were controlled as well [71,88]. DCD was found to yield compost with a good maturity index, which could further verify the feasibility of applying DCD [100]. As more dicyandiamide is used in combination with other additives, it will be analyzed and discussed in the section on compound additives.

#### 3.3.4. Cost Assessment and Economic Benefits

The costs of chemical additives are relatively low and the supply channel is more stable than that of physical additives. Indeed, the impacts of chemical additive residues on crop and compost efficiency need to be further verified and included in the economic benefit assessment.

**Table 3:** The effects of chemical additives on GHGs and odors during composting.

Feedstock	Additive	Impact on Gaseous Emissions (Relative to Control)				Note	Reference
		CH_4_	N_2_O	NH_3_	VSCs		
Pig manure, corn stalk	0.2% dicyandiamide ^2^	−20%	−32%	−9%		Lasted for 40 days; similar temperature variation initial C/N: 20	[24]
Pig manure, corn stalk	1.5% ferric oxide ^1^				↓	Lasted for 14 days; promoted temperature rise; reduced volatile sulfur compounds by 46.7–80.8%	[17]
Goat manure, wheat straw	8 mg/kg sodium selenite ^1^		−70%	↓		Lasted for 80 days; similar temperature variation	[13]
Goat manure, wheat straw	2 mg/kg sodium selenite ^2^	+18%	−30%	−27%		Lasted for 80 days; similar temperature variation	[91]
	2 mg/kg sodium selenate ^2^	+4%	−62%	−53%			
Poultry manure, sawdust	H_2_SO_4_ (pH = 6)	−20%	−18%	−21%		Lasted for 42 days; later, but higher, temperature peak	[71]
Chicken manure, tobacco waste	5% calcium superphosphate ^2^	↓	−79%	−37%		Lasted for 35 days; promoted temperature rise	[64]
Dewatered sewage sludge, sawdust	5% magnesium chloride ^2^	−23%		−59%		Lasted for 23 days; promoted temperature rise	[85]
	5% ferrous sulfate ^2^	−25%	+ 16%	−83%			
Rice husk chicken manure, slaughter sludge	1% lactic acid ^2^			−33%		Lasted for 28 days; promoted temperature rise	[2]
Dewatered sewage sludge, wheat straw	1% citric acid ^2^	−33%	−51%	↓		Lasted for 45 days; similar temperature variation	[22]
	1% elemental sulfur ^2^	−71%	+48%	↓			
	3% phosphoric acid ^2^	−53%	+32%	↓			
	3% magnesium hydrogen phosphate ^2^		−70%	↓			
	5% calcium superphosphate ^2^	−43%	+15%	↓			
Sewage sludge, corn stalk	10% phosphogypsum ^2^	−81%	↑	−17%		Lasted for 35 days; later temperature peak	[82]
	10% superphosphate ^2^	−75%	−55%	−36%			
Chicken manure, mushroom residue	0.25% sulfur powder (net weight)			↓		last for 21 days	[101]
Pig manure, woody peat	10% calcium superphosphate ^2^	−36%	↑	−38%	↓	Lasted for 28 days; suppressed temperature rise; reduced H_2_S by 66%	[83]
Pig manure, corn stalk	15% H_3_PO_4_ ^3^, 15% Mg (OH)_2_ ^3^			↓		Lasted for 35 days; similar temperature variation	[81]
	15% KH_2_PO_4_ ^3^, 15% MgSO_4_ ^3^	↓		↓			
	15% Ca(H_2_PO_4_)_2_ ^3^, 15% MgSO_4_ ^3^	↓		↓			
	15% H_3_PO_4_ ^3^, 15% MgSO_4_ ^3^	↓		↓			

Note: ^1^ wet weight basis; ^2^ dry weight basis; ^3^ molar ratio of initial nitrogen; ↑: increase (no detailed data); ↓: decrease (no detailed data); VSCs: volatile sulfide compounds.

### 3.4. Microbial Additives

#### 3.4.1. Efficiency Analysis

Microbial additives inoculate dominant bacteria in the compost, mainly in the form of bacterial agents, skipping the generation of natural succession, and they can significantly accelerate the maturation process and enhance microbial activity [102]. The microbial community structure of compost was changed by inoculation with a bacterial agent, and adverse bacteria were antagonized to achieve the purpose of inhibiting the loss of nutrients in the form of gases [103]. As shown in Table 4, the addition of bacterial agents as microbial additives mainly corresponded to the required composting stage; the composting process could be influenced by the regulation of the microbial community’s structure. According to the research by Xue et al. [19], an aerobic microorganism agent significantly reduced CH_4_ emissions. Inoculation with aerobic bacteria enhanced the mineralization intensity during the thermophilic period. With the accumulation of ammonium nitrogen, the activity of methanogens was inhibited, and CH_4_ emissions were significantly reduced [88]. The CH_4_ reduction performance of aerobic microbial inoculation was also reported by Gao et al. [23], but the emissions of N_2_O were reduced. Xie et al. [104] also found that ammonia-oxidizing archaea reduced N_2_O emissions. The addition of oxidizing bacteria could significantly activate native microbial communities in compost, exhibiting an earlier entrance into the thermophilic period, a higher peak temperature, and a longer duration of the thermophilic period [105]. However, the higher microbial activity induced rapid changes in the physical properties of a composting pile, which was the main reason for the generation of an anaerobic area during the thermophilic period [106]. It can be concluded that the capacity of a composting pile to hold NH_4_^+^ and NO_2_^−^ is limited. When the microbial metabolism intensity exceeds the reactor capacity, incomplete nitrification/denitrification occurs, and N_2_O emissions increase [107]. In contrast, an appropriate metabolic intensity beneath the concentration limit can provide a more comprehensive reduction in emissions [23]. Zhao et al. [108] isolated thermotolerant nitrifying bacteria (TNB) enriched in compost as a microbial agent. The TNB treatment promoted the nitration reaction and the conversion of ammonium to nitrate, reducing NH_3_ emissions by 29.7%. Additionally, Chen et al. [109] observed that thermotolerant sulfide-oxidizing inoculants reduced NH_3_ and H_2_S emissions by 19.4% and 48.9%, respectively. It can be concluded that thermophilic/thermostable bacterial inoculants provide a more stable metabolic function in the thermophilic phase. TNB alleviate NH_3_ emissions through more intense nitrification [108]. Otherwise, thermotolerant sulfide-oxidizing inoculants inhibit the generation of H_2_S by guiding efficient oxidation of its precursors, and metabolic acidification also alleviates NH_3_ emissions [20,109]. Wang et al. [110] and Kuroda et al. [111] reported that *Bacillus* inoculation could effectively reduce NH_3_ emissions. Qiu et al. [112] found that a nitrogen-retaining microbial agent also mitigated NH_3_ emissions. As most of the bacterial agents added in experiments have been compound-specific bacteria prepared after separation and purification, no gas emission trend with an insignificant impact is recorded in Table 4. Furthermore, the effect of combined microbial agents and other additives will be further discussed in the following section about compound additives.

#### 3.4.2. Cost Assessment and Economic Benefits

At present, most bacterial agents on the market are used to accelerate compost maturation, and their price is moderate. However, GHG emissions cannot be controlled specifically due to their composition. Therefore, bacterial agents that are more effective in controlling GHG emissions need to be further developed while reducing costs and improving economic benefits.

### 3.5. Compound Additives

#### 3.5.1. Efficiency Analysis

Currently, there is increased research interest in using a variety of additives and technological processes to comprehensively control the loss of nutrients in the form of gaseous products. By enhancing the fixation of nutrients, it is possible to improve the quality of compost and the feasibility of its production.

Compared with solely electric field-assisted treatment, biochar combined treatment could further reduce CH_4_ and N_2_O emissions by 69.58% and 31.16% (compared with an untreated control), respectively [21]. The pore structure of biochar provides a higher oxygen supply rate for a composting pile driven by the electric field, enhances microbial metabolism intensity, and changes the community structure [113]. Wang et al. [114] used biochar with wood vinegar in a combined treatment and reduced the CH_4_, NH_3_, and N_2_O emissions by 62.75%, 35.85%, and 24.61%, respectively. As an organic physical additive, biochar provides additional aeration properties and does not affect composting via other reactions outside the carbon source. The addition of wood vinegar further reduced the release of NH_3_ by regulating the pH environment, and a high concentration of NH_4_^+^ was also well-adsorbed by biochar [115]. Therefore, biochar and acidic chemical additives exhibit a combined and synergistic effect. Besides the combination of biochar and chemical additives, adding microbial agents is also an excellent combined treatment. Xue et al. [19] proved that combining different microbial agents and biochar could further enhance the reduction in GHG emissions (Table 5). Based on biochar modification, the combined treatment provided a larger space and a broader oxygen supply channel for vigorous life activities after inoculation [66]. It also avoided the occurrence of incomplete nitrification and denitrification [116]. However, the combination of mineral materials and acidic additives is prone to chemical reactions, which limits the application of this approach. Awasthi et al. [117] used biochar and calcium bentonite as compound additives. The combined treatment was shown to reduce CH_4_ and N_2_O emissions more than sole biochar addition, but resulted in higher NH_3_ emissions. The same result was obtained in their earlier research [118]. As mineral additives, lime and zeolite behave like typical physical additives, providing improved pore structure to a composting pile and allowing microbial aerobic respiration [73]. An increase in NH3 volatilization may be due to the alkalization of the composting pile by dissolved lime [119]. Alkaline environments enhance the conversion of unstable ammonium nitrogen to NH_3_, which can be attributed to other mineral additives, such as montmorillonite [120]. The reactions of mineral additives are usually accompanied by exothermic heat release. In addition, high treatment temperatures are not conducive to NH_3_ retention [20,85]. Thus, the combination of different physical additives may provide better performance toward the mitigation of GHGs.

As mentioned above, dicyandiamide (DCD) is a harmless and mature nitrification inhibitor for the composting process. Yang et al. [24] and Yuan et al. [82] conducted similar studies on the combined effect of DCD and other phosphorus compounds. Jiang et al. [121] reported that a “dicyandiamide + thiophosphoric triamide” treatment significantly mitigated the emissions of N_2_O and NH_3_ by 63.9% and 26.3%, respectively. The phosphorus additive and DCD caused stronger accumulation of nitrogen in the form of ammonium nitrogen, and the combined treatments exhibited a higher NH_4_^+^ content. As mentioned in the analysis of the single effect of chemical additives, a higher NH_4_^+^ content is conducive to the inhibition of methanogens, which explains the superposition of the methane emission reduction effect in the combined treatment [89]. Under the action of DCD, the conversion pathway of ammonium nitrogen to nitrite nitrogen is blocked, which promotes the existence of more nitrogen in the form of ammonium nitrogen and significantly reduces the emissions of N_2_O [122]. Generally, by adjusting the pH or generating struvite crystallization, ammonium nitrogen can be stably preserved and NH_3_ emissions can be reduced [71,100]. In the case of NH_3_ volatilization, the higher content of ammonium nitrogen under the combined treatment needs to be stabilized [66]. According to the inference, it can be concluded that the upper limit of ammonium fixation is determined by the internal physical properties of a composting pile and the addition of physical additives, such as biochar, can increase its capacity so the dynamic equilibrium of ammonium nitrogen is more favorable to nitrogen fixation [74,107]. After the use of a higher dose of DCD or its combined treatment, the mineralization extent is increased, the nitrification pathway is completely cut off, and the ammonium nitrogen that cannot be stably adsorbed only exists in the form of NH_3_ with forced ventilation [82,115].

The remaining compound additives are basically reacted as a mixture. Liu et al. [67] added a compound fertilizer that contained calcium, magnesium, and phosphorus to compost. It reduced the emissions of NH_3_ by 42.9%, H_2_S by 34.91%, Me_2_S by 100%, and Me_2_SS by 63.28%. CaSO_4_⋅H_2_O and free phosphoric acid in compost can effectively adsorb NH_4_^+^ [67]. Struvite crystallization is also conducive to the fixation of more ammonium nitrogen [96]. Although NH_3_ emissions were significantly reduced in this research, a higher concentration of free NH_4_^+^ could potentially lead to an increase in NH_3_ emissions, which also depended on the adsorption capacity of the pile [97]. The generation of odors mainly originates from the anaerobic decomposition of proteins [38]. As Liu et al. [67] reported, the main odor components in volatile sulfur compounds are Me_2_S and Me_2_SS, and they are formed by the methylation of H_2_S. Fertilizer compound treatment is more helpful in reducing the formation of odor precursors to weaken methylation and alleviate odor emissions.

Chen et al. [123] specifically focused on chicken manure composting and employed wheat straw as a bulking agent. Compound treatment mitigated NH_3_, N_2_O, and CH_4_ emissions by 41.4%, 9.0%, and 55.9%, respectively. A direct positive correlation was found between the amount of the compound additive added and the extent of the reduction in NH3 and CH4 emissions. Because of the high temperature formed due to the compound additive, nitrifying bacteria and methanogens were strictly inhibited [76,124]. Under the combined action of biochar and inoculated microorganisms, the compost maintained good metabolic efficiency, anaerobic areas were rarely produced, and N_2_O and CH_4_ emissions were greatly reduced [125]. As discussed in Section 3.4, the strong microbial activity and high NH_4_^+^ content that form at higher temperatures require stronger adsorption and fixation capabilities. The aeration conditions provided by 10% chicken manure biochar (CMB) were matched by a 10% chicken manure integrated microbial consortium (CMMC). Thus, nitrogen was mostly fixed in the form of ammonium nitrogen, and the emissions of NH_3_ were effectively reduced [115].

The application of mature compost has recently become a hot topic in the composting field [126]. The essence of mature compost is the backfill of finished compost products, which helps to reduce the cost under continuous production conditions [126,127]. Although backfilled mature compost undergoes certain nutrient loss, its use as an additive can provide a pore structure and microbial community that raw materials cannot reach [128]. Yang et al. [129] reported that the addition of mature compost could reduce the emissions of NH_3_, N_2_O, and CH_4_ by 58.0%,73.6%, and 44.8%, respectively. In a sense, mature compost is equivalent to the inoculation of a physical additive and a microbial agent. Mixing mature compost provides the composting pile with stronger microbial activity and an aeration effect [130]. Similar to the study by Chen et al. [109], the same theory can explain the reduction in CH_4_ and N_2_O emissions. Mixing highly humified mature compost is conducive to enhancing the adsorption of ammonium nitrogen in a composting pile, which can significantly reduce ammonia volatilization [131,132]. Luo et al. [133] observed almost the same trend of mature composting reducing GHG emissions, but the NH_3_ emission reduction performance was not sufficiently explained. Different treatment methods of mature compost lead to different emission reduction effects when used as an additive. Finally, it is more effective to use a mixed treatment for mature compost addition [129].

**Table 5:** The effects of compound additives on GHGs and odors during composting.

Feedstock	Additive	Impact on Gaseous Emissions (Relative to Control)				Note	Reference
		CH_4_	N_2_O	NH_3_	VSCs		
Chicken manure, rice husk	electric field-assisted composting (2 V) +10% biochar ^1^	−70%	−31%			Lasted for 30 days; promoted temperature rise; increased electrical conductivity	[21]
Pig manure, apple sawdust	5% biochar ^2^ + 1.0% wood vinegar ^2^	−63%	↓	−36%		Lasted for 64 days; similar temperature variation	[114]
Sewage sludge, straw	5% bamboo biochar ^1^ +0.3% aerobic microorganism agent ^1^	−45%	↓			Lasted for 29 days; promoted temperature rise	[19]
	5% bamboo biochar ^1^ +0.3% facultative anaerobic microorganism agent ^1^	−44%	↓				
Dewatered sewage sludge, wheat straw	12% wheat straw biochar ^2^ + 4% calcium-bentonite ^2^	↓	↓	↑		Lasted for 43 days; promoted temperature rise	[117]
	30% zeolite ^2^ + 1% lime ^2^	↓	↓	↑		Lasted for 56 days; promoted temperature rise	[118]
Pig manure, corn stalk	0.2% dicyandiamide ^2^ + 6.6% phosphogypsum ^2^	−39%	−36%	−18%		Lasted for 40 days; similar temperature variation; initial C/N: 17, 16; increased electrical conductivity	[24]
	0.2% dicyandiamide ^2^ + 6.6% superphosphate ^2^	−33%	−25%	−21%			
Pig manure, sawdust	0.5% thiophosphoric triamide ^2^ +0.25% dicyandiamide ^2^		−64%	−27%		Lasted for 20 days; similar temperature variation	[121]
Sewage sludge, corn stalk	10% phosphogypsum ^2^ + 2.5% dicyandiamide ^3^	↓	−86%	+20%		Lasted for 35 days; later temperature peak; both reduced CH_4_ emissions by more than 50%	[82]
	10% superphosphate ^2^ + 2.5% dicyandiamide ^3^	↓	−88%	−12%			
Pig manure, corn stalk	10% calcium magnesium phosphate fertilizer ^2^		↑	−43%	↓	Lasted for 49 days; promoted temperature rise; H_2_S: –35%; Me_2_S: –100%; Me_2_SS: –63%	[67]
Chicken manure, wheat straw	10% chicken manure integrated microbial consortium ^2^ + 10% chicken manure biochar ^2^	−56%	−9%	−41%		Lasted for 42 days; similar temperature variations	[123]
Kitchen waste, corn stalk	10% mature compost (mix) ^1^	−45%	−74%	−58%		Lasted for 35 days; promoted temperature rise	[129]
Pig manure, corn stalk	5% mature compost (cover) ^1^	−59%	↓	+61%		Lasted for 30 days; promoted temperature rise; all reduced N_2_O emissions significantly	[133]
	5% mature compost (mix) ^1^	−53%	↓				
	5% mature compost (cover + mix) ^1^	−65%	↓				

Note: ^1^ wet weight basis; ^2^ dry weight basis; ^3^ molar ratio of initial nitrogen; ↑: increase (no detailed data); ↓: decrease (no detailed data); VSCs: volatile sulfide compounds.

#### 3.5.2. Cost Assessment and Economic Benefits

Compound additives include “process + additives” and “additive-combined treatment”, leading to higher costs than those incurred in the case of single measures. However, compared with single measures, combined treatments exhibit better performance in reducing GHGs and odors [19,21,24]. The specific economic benefits should be evaluated in accordance with the actual situation.

## 4. Prospective

Based on the review of different additives and their reaction modes, the appropriate use of additives can effectively accelerate the composting period, improve product quality, and reduce air pollution. In particular, mineral additives are expensive and cannot be recycled, so they are not the first choice for production applications. Mineral additives and chemical additives trigger chemical reactions in a composting pile and release ions, reflected as increased electrical conductivity, and may increase the toxic effect on crops [134]. Although organic physical additives have lower costs and considerable efficiency, their preparation and supply restrict the production scale [67,123]. Similarly, the current composting process and microbial additives are mainly used to accelerate maturation, and they lack pertinence for gaseous emission reductions during composting [108,109]. Also, processes such as membrane-covered composting and electric field-assisted composting are expensive for large-scale production [58,60]. A comprehensive consideration of cost and economic benefits will help to improve production feasibility and facilitate long-term development. With the promotion of composting and the expansion of the production scale, it is necessary to conduct stricter assessments of cleaner production and more complete quantitative gas emission monitoring in the whole process. Therefore, the future prospectives for GHG and odor reduction during composting are as follows:

1. Before composting: more low-cost and recyclable materials should be developed as additives; continuous and stable supply will be an important criterion for judging additives;
2. During composting: the use of microbial agents should be more targeted, not only for accelerating maturation, but also for screening highly efficient strains that are more conducive to GHG and odor emissions reduction;
3. After composting: expand from the laboratory scale to industrial production; continuously and quantitatively monitor GHGs and odor emissions, and unify the measurement method and magnitude; ensure strict control of GHGs and odor emissions throughout the process, from compost production to crop planting.

## 5. Conclusions

This study summarizes the influence of composting conditions and different additives on gaseous emissions. Greenhouse gases can be effectively reduced by adjusting composting conditions. Anaerobic gaseous products can be diminished by the application of physical porous additives. Chemical additives significantly reduce gaseous emissions, but their side effects on compost application must be eliminated. The reduction effects of microbial additives are influenced by the addition amounts and their microenvironment. Compound additives can further reduce gaseous emissions. However, further studies are required to assess the economic viability of additives to promote their large-scale application during composting.

## Figures and Tables

**Table 4:** The effects of microbial additives on GHGs and odors during composting.

Feedstock	Additive	Impact on Gaseous Emissions (Relative to Control)				Note	Reference
		CH_4_	N_2_O	NH_3_	VSCs		
Sewage sludge, straw	0.3% aerobic microorganism agent ^1^	−25%	↑			Lasted for 29 days; promoted temperature rise	[19]
	0.3% facultative anaerobic microorganism agent ^1^	−8%	↑				
Kitchen waste, garden waste	0.9% aerobic microbial inoculant ^1^	↓	↓	↑	↑	Lasted for 35 days; promoted temperature rise	[23]
Sewage sludge, rice husk	5% thermotolerant sulfide-oxidizing compound bacterial consortium (*v/w*)			−19%	↓	Lasted for 22 days; promoted temperature rise; reduced H_2_S by 49%	[109]
Sewage sludge, spent mushroom substrate	5% thermophilic nitrifying bacteria (*v/w*)			↓		Lasted for 20 days; promoted temperature rise; initial C/N: 16	[108]
Chicken manure, rice husks	10% nitrogen-retaining microbial agent ^1^			↓		Lasted for 45 days; promoted temperature rise	[112]
Poultry manure, sawdust	*Bacillus* stearothermophilus (8 g/kg)			↓		Lasted for 12 days; similar temperature variation; significantly reduced NH_3_ emissions	[110]
Pig manure, sawdust	*Bacillus* sp.			↓		Lasted for 18 days; similar temperature variations	[111]
Chicken manure, rice husk, bran, mushroom residue	5% ammonia-oxidizing archaea (*w/v*)			↓		Lasted for 45 days; promoted temperature rise; initial C/N: 32; significantly reduced NH_3_	[104]

Note: ^1^ wet weight basis; ↑: increase (no detailed data); ↓: decrease (no detailed data); VSCs: volatile sulfide compounds.

## Data Availability

We conducted secondary data analysis of publicly available data.
